# Publisher Correction: A *PRDX1* mutant allele causes a *MMACHC* secondary epimutation in *cblC* patients

**DOI:** 10.1038/s41467-018-03054-w

**Published:** 2018-02-02

**Authors:** Jean-Louis Guéant, Céline Chéry, Abderrahim Oussalah, Javad Nadaf, David Coelho, Thomas Josse, Justine Flayac, Aurélie Robert, Isabelle Koscinski, Isabelle Gastin, Pierre Filhine-Tresarrieu, Mihaela Pupavac, Alison Brebner, David Watkins, Tomi Pastinen, Alexandre Montpetit, Fadi Hariri, David Tregouët, Benjamin A Raby, Wendy K. Chung, Pierre-Emmanuel Morange, D. Sean Froese, Matthias R. Baumgartner, Jean-François Benoist, Can Ficicioglu, Virginie Marchand, Yuri Motorin, Chrystèle Bonnemains, François Feillet, Jacek Majewski, David S. Rosenblatt

**Affiliations:** 1 INSERM, UMR_S954 Nutrition-Genetics-Environmental Risk Exposure and Reference Centre of Inborn Metabolism Diseases, University of Lorraine and University Hospital Centre of Nancy (CHRU Nancy), 54505 Nancy, France; 2 Department of Human Genetics, McGill University and Research Institute McGill University Health Centre, H4A 3J1 Montreal, Quebec Canada; 3 Sorbonne Universités, UPMC University Paris 06, Institut National pour la Santé et la Recherche Médicale (INSERM), ICAN Institute for Cardiometabolism and Nutrition, Unité Mixte de Recherche en Santé (UMR_S) 1166, Team Genomics & Pathophysiology of Cardiovascular Diseases, 75013 Paris, France; 4 Channing Division of Network Medicine, Department of Medicine, Brigham and Women’s Hospital, Harvard Medical School, 02115 Boston, MA United States of America; 5 Departments of Pediatrics and Medicine, Columbia University, 10032 New York, NY United States of America; 6 INSERM, UMR_S1062, Nutrition Obesity and Risk of Thrombosis, Aix-Marseille University, 13005 Marseille, France; 7 Division of Metabolism and Children’s Research Centre (CRC), University Children’s Hospital, CH-8032 Zürich, Switzerland; 8 Service de Biochimie Hormonologie, Hôpital Robert Debré, 75019 Paris, France; 9 Children’s Hospital of Philadelphia, Perelman School of Medicine at the University of Pennsylvania, 19104 Philadelphia, PA United States of America; 10 Laboratoire Ingénierie Moléculaire et Physiopathologie Articulaire (IMoPA), UMR7365 CNRS - Université, de Lorraine and FR3209 CNRS- Université de Lorraine, 54505 Nancy, France

Correction to: *Nature Communications* 10.1038/s41467-017-02306-5, published online 4 January 2018

The original version of this Article contained an error in the title, which was incorrectly given as “APRDX1 mutant allele causes a *MMACHC* secondary epimutation in *cblC* patients”. This has now been corrected in both the PDF and HTML versions of the Article to read “A *PRDX1* mutant allele causes a *MMACHC* secondary epimutation in *cblC* patients”.

