# Supplementary material for: A PRDX1 mutant allele causes a MMACHC secondary epimutation in cblC patients
Source: Nat Commun. 2018 Jan 4;9:67. doi: 10.1038/s41467-017-02306-5 (PMC5754367; doi:10.1038/s41467-017-02306-5)
Supplement: Supplementary file 1 — Supplementary Information [file 41467_2017_2306_MOESM1_ESM.pdf]

41467_2017_2306_MOESM1_ESM.pdf: Supplementary Figure and Tables of the article "A PRDX1 mutant allele causes a MMACHC secondary epimutation in cblC patients", DOI 10.1038/s41467-017-02306-5

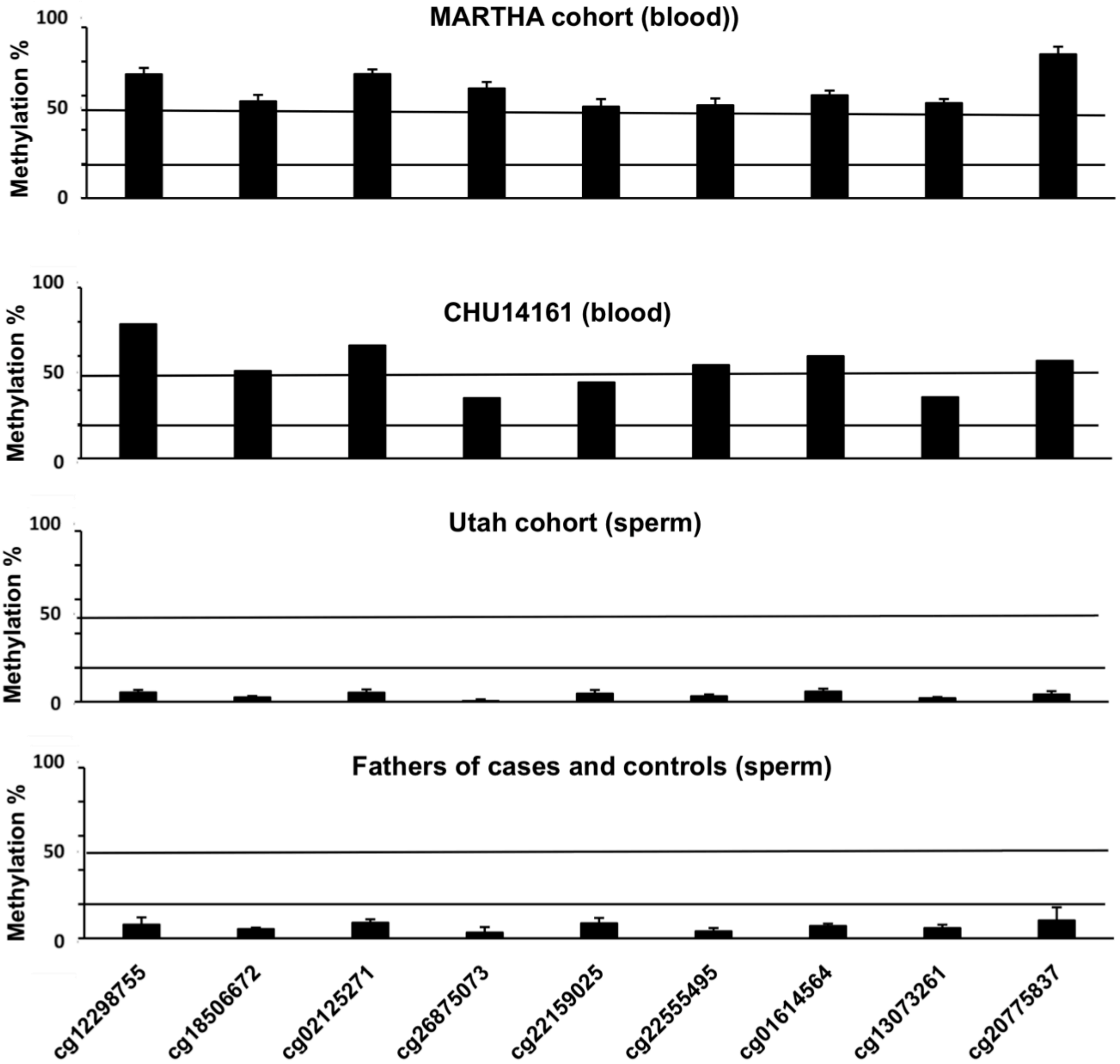

Supplementary Figure 1. Methylation analysis of SNRPN imprinted gene in sperm from proband's fathers. The absence of contamination of sperm DNA by somatic DNA was proven by methylation analysis of *SNRPN* imprinted gene. As expected, *SNRPN* was unmethylated in sperm DNA from the fathers and two paired controls and UTAH cohort and methylated in the blood DNA from the MARTHA cohort (mean +/- standard deviation of beta values) and from the father of case 12122 (CHU14161). The analysis is based on β-values obtained from the Infinium HumanMethylation450 Bead Chip array. The line corresponds to a β value threshold of 0.2, below which the CpG probe was considered fully unmethylated.

Supplementary Table 1: *Epi-CblC* cases and their relatives with *MMACHC* monoallelic coding mutation, *MMACHC* epimutation and *PRDX1 c.515-1G>T* mutation (ND: not determined)

Identification code	Proband/ relatives	MMACHC heterozygous mutation	Epimutation in MMACHC	PRDX1 c.515-1G>T	Antisense lncRNA*	Age	Gender	Ethnicity/ country
MG-002151	Case	*c.617G>A* (p.Arg206Gln)	yes	GT	Yes	63 years	M	Caucasian /Italy
MG-002153	Case	*c.271dupA* (p.Arg91LysfsX14)	yes	GT	Yes	3 weeks	F	Caucasian /Australia
F-R1	Case	*c.271 dupA*	yes	GT	Yes	6 years	M	Caucasian /France
F- R11	Father F-44744	*wt*	yes	GT	ND	40 years	M	Caucasian /France
F-R12	Mother F-44744	*c.271 dupA*	no	GG	ND	33 years	F	Caucasian /France
F-R2	Case	*c.481C>T (p.Arg161X)*	yes	GT	Yes	8 years	F	Caucasian /France
F-R21	Father F-40039	*c.481C>T (p.Arg161X)*	no	GG	ND	42 years	M	Caucasian /France
F-R22	Mother F-40039	*wt*	yes	GT	ND	32 years	F	Caucasian /France
F-R3	Case	*c.271 dupA*	yes	GT	ND	5 years	F	Caucasian /France
F-R31	Father F-50747	*c.271 dupA*	no	GG	ND	39 years	M	Caucasian /France

Supplementary Table 2. List of genes reverse (R1)-forward (F2)-reverse (R3) gene trios, with recessive transmission of inherited diseases produced by R2 gene mutations and Triplex Target Sites (TTS) and CpG island in R1/F2 bidirectional promoter

R1 reverse gene	F2 forward gene	R3 reverse gene	CpG island, R1-F2	CpG island R1-F2 (methylation status)	OMIM-R1 (OMIM#)	OMIM-F2 (OMIM#)	OMIM-R3 (OMIM#)
CCDC163P †	*MMACHC **	*PRDX1*	CpG: 33	Unmethylated	Non-Morbid	Methylmalonic aciduria and homocystinuria, cblC type, (277400)	Non-Morbid
AUP1	*HTRA2**	*LOXL3*	CpG: 153	Unmethylated	Non-Morbid	Parkinson disease 13, 610297 (3)	Non-Morbid
CIDECP †	*FANCD2**	*FANCD2OS*	CpG: 41	Unmethylated	Not referenced	Fanconi anemia, complementation group D2	Not referenced
UBE2D3	*CISD2**	*SLC9B1*	CpG: 141	Unmethylated	Not referenced	Wolfram syndrome 2, (604928)	Non-Morbid
ENC1	*HEXB**	*GFM2*	CpG: 160	Unmethylated	Non-Morbid	Sandhoff disease, infantile, juvenile, and adult forms, 268800 (3); Spinal muscular (atrophy, juvenile)	Non-Morbid
MGAT4B	*SQSTM1**	*C5orf45*	CpG: 193	Unmethylated	Non-Morbid	Paget disease of bone, (602080)	Not referenced
LOC100129518 ‡	*ACAT2**	*TCP1*	CpG: 105	Unmethylated	Not referenced	ACAT2 deficiency (1)	Non-Morbid
MCM7	*AP4M1**	*TAF6*	CpG: 141	Unmethylated	Non-Morbid	Cerebral palsy, spastic quadriplegic, 3, (612936)	Non-Morbid
LOC101927612 ‡	*RNF139**	*TATDN1*	CpG: 92	Unmethylated	Not referenced	Renal cell carcinoma, (144700)	Not referenced
DUOX2*	*DUOXA2**	*DUOXA1*	CpG: 35	Unmethylated	Thryoid dyshormonogenesis 6, 607200 (3)	Thyroid dyshormonogenesis 5, 274900	Non-Morbid
TIPIN	*MAP2K1**	*SNAPC5*	CpG: 114	Hemimethylated	Non-Morbid	Cardiofaciocutaneous syndrome, (115150)	Non-Morbid
TSR3	*GNPTG**	*UNKL*	CpG: 122	Unmethylated	Not referenced	Mucolipidosis III gamma, (252605)	Not referenced
NTHL1	*TSC2**	*PKD1**	CpG: 94	Unmethylated	Non-Morbid	Lymphangioleiomyomatosis, somatic, (606690) ,Tuberous sclerosis-2, (191100)	Polycystic kidney disease, adult type I, (173900)
SLX4	*DNASE1**	*TRAP1*	CpG: 67	Unmethylated	Not referenced	{Systemic lupus erythematosus, susceptibility to}, (152700)	Non-Morbid
VPS9D1	*ZNF276*	*FANCA**	CpG: 227	Unmethylated	Not referenced	Non-Morbid	Fanconi anemia, complementation group A, (227650)
SHPK	*CTNS**	*P2RX5-TAX1BP3*	CpG: 114	Unmethylated	Non-Morbid	Cystinosis, nephropathic, (219800); Cystinosis, ocular nonnephropathic, (219750); Cystinosis, late-onset juvenile or adolescent nephropathic, (219900); Cystinosis, atypical nephropathic	Non-Morbid
MEF2BNB	*RFXANK**	*NR2C2AP*	CpG: 30 + CpG: 19	Unmethylated	Not referenced	MHC class II deficiency, complementation group B, (209920)	Non-Morbid
ADCK4 ‡	*ITPKC**	*C19orf54*	CpG: 56	Unmethylated	Not referenced	{Kawasaki disease, susceptibility to}, (611775)	Not referenced
NEURL2	*CTSA**	*PLTP**	CpG: 141	Unmethylated	Non-Morbid	Galactosialidosis	[High density lipoprotein cholesterol level QTL 9]

NOTE. R1: Reverse-1; F2: Forward-2; R3: Reverse-3; OMIM: Online Mendelian Inheritance in Man; TTS: triplex target DNA sites; *Genes with pathogenic annotation on OMIM database; † Pseudogene; ‡ Uncharacterized

Supplementary Table 3. Primers used in PCR, RT-PCR and qRT-PCR experiments

Application	Primer Name	Direction	Primer sequences 5'→3'
Bisulfite converted DNA PCR	MMACHC_BS_F	forward	TTAAATTTGTGTTAGTGATAATTGT
Bisulfite converted DNA PCR	MMACHC_BS_R	reverse	AACTAACCTAAAAAAAATAAACCTC
RT-PCR	MMACHC_cDNA_F	forward	CAGCAAGCTCAGCGTGTAAC
RT-PCR	MMACHC_cDNA_R	reverse	CCACCATAAATCAGGGTCCA
RT-PCR	PRDX1_S	forward	CATTCCTTTGGTATCAGACCCG
RT-PCR	CCDC163P_AS	reverse	AGCGTTGAGAAGCACATCCA
qRT-PCR	MMACHC	forward	ATCTGGGCCGTGTTAGAGAGA
qRT-PCR	MMACHC	reverse	CCTCCACATCTTGTCGTTGG
qRT-PCR	*PRDX1*	forward	CCACGGAGATCATTGCTTTCA
qRT-PCR	*PRDX1*	reverse	AGGTGTATTGACCCATGCTAGAT
qRT-PCR	*POLR2A*	forward	CAGACCGGCTATAAGGTGGA
qRT-PCR	*POLR2A*	reverse	GGTAGACCATGGGAGAATGC
